# Increased Cytosolic Calcium Contributes to Hydrogen-Rich Water-Promoted Anthocyanin Biosynthesis Under UV-A Irradiation in Radish Sprouts Hypocotyls

**DOI:** 10.3389/fpls.2018.01020

**Published:** 2018-07-16

**Authors:** Xiaoyan Zhang, Junyu Wei, Yifan Huang, Wenbiao Shen, Xin Chen, Chungui Lu, Nana Su, Jin Cui

**Affiliations:** ^1^College of Life Sciences, Nanjing Agricultural University, Nanjing, China; ^2^Institute of Industrial Crops, Jiangsu Academy of Agricultural Sciences, Nanjing, China; ^3^School of Animal, Rural and Environmental Sciences, Nottingham Trent University, Nottingham, United Kingdom

**Keywords:** calcium, hydrogen-rich water, radish sprouts, anthocyanin, UV-A

## Abstract

Our previous studies showed that hydrogen-rich water (HRW) promoted the biosynthesis of anthocyanin under UV-A in radish. However, molecular mechanism involved in the regulation of the anthocyanin biosynthesis is still unclear. In this study, the role of calcium (Ca^2+^) in HRW-promoted anthocyanin biosynthesis in radish sprouts hypocotyls under UV-A was investigated. The results showed that a positive effect of HRW on the content of cytosolic calcium and anthocyanin accumulation, mimicking the effects of induced CaCl_2_. Exogenous addition of Ca^2+^ chelator bis (β-aminoethylether)-N,N,N′,N′-tetraacetic acid (EGTA) and inositol 1,4,5-trisphosphate (IP3) synthesis inhibitor neomycin partially reversed the facilitated effect of HRW. The positive effects of HRW on activity of anthocyanin biosynthetic-enzymes (*L*-phenylalanine ammonia-lyase, PAL; chalcone isomerase, CHI; dihydroflavonol 4-reductase, DFR and UDP glc-flavonoid 3-*O*-glucosyl transferase, UFGT) were reversed by EGTA and neomycin. Further tests confirmed that the upregulation of anthocyanin biosynthetic related genes induced by HRW was substantially inhibited by calcium antagonists. The possible involvement of CaM in HRW-regulated anthocyanin biosynthesis was also preliminarily investigated in this study. Taken together, our results indicate that IP3-dependent calcium signaling pathway might be involved in HRW-regulated anthocyanin biosynthesis under UV-A irradiation.

## Introduction

Anthocyanins, belonging to flavonoids, are naturally occurring water-soluble pigments which confer red, orange, blue, and purple colors in fruits and vegetables (Harborne and Williams, 2000; Jaakola, 2013). In plants, anthocyanins play important physiological roles, such as scavenging free radicals, attracting insect pollination, and resisting UV radiation (Harborne and Williams, 2000; Nijveldt et al., 2001; Glover and Martin, 2012). Anthocyanins can also act as antibacterial agents against bacteria invasion (Harborne and Williams, 2000). In addition, numerous studies have shown that anthocyanins have antioxidant, anti-inflammatory, and anti-tumor-promoting effects (Kong et al., 2003; Nam et al., 2005; Xia et al., 2006; Pascual-Teresa and Sanchez-Ballesta, 2008; Santosbuelga et al., 2014). Consuming anthocyanin-rich foods can significantly reduce the risk of chronic diseases in humans (Toufektsian et al., 2008; Abdenour and Charles, 2014; Ali et al., 2017). Therefore, the study of promoting anthocyanin biosynthesis in crops has potential to significantly impact human nutrition and health.

The biosynthesis pathway of anthocyanins is well studied in model plant *Arabidopsis* (Koes et al., 2005; Gonzalez et al., 2008; Jaakola, 2013). Anthocyanins are synthesized via the phenylpropanoid pathway and the key enzymes include phenylalaninammo-nialyase (PAL), chalcone synthase (CHS), chalcone isomerase (CHI), flavanone 3-hydroxylase (F3H), flavonoid 3′-hydroxylase (F3′H), dihydroflavonol 4-reductase (DFR), leucoanthocyanidin dioxygenase (LDOX), and UDP glc-flavonoid 3-*O*-glucosyltransferase (UFGT) (Gonzalez et al., 2008; Jaakola, 2013). Transcriptional control of anthocyanins biosynthesis has been studied in *Arobidopsis* toward understanding and manipulating color phenotype. WD-repeat/bHLH/MYB (MBW) complex, which was composed of TRANSPARENT TESTA GLABRA 1 (TTG1), basic-helixloop-helix (bHLH) transcription factors, and R2R3 MYB transcription factors, plays a major regulatory role in regulating anthocyanin biosynthesis (Koes et al., 2005; Gonzalez et al., 2008; Xu et al., 2015). PRODUCTION OF ANTHOCYANIN PIGMENT (PAP) protein family of R2R3 MYB transcription factors are specifically needed for anthocyanin biosynthesis and are therefore believed to be key components of MBW complex (Borevitz et al., 2000; Shi and Xie, 2010). The biosynthesis of anthocyanins in plants is regulated by both developmental signals and environmental signals, such as plant hormones, UV, high light intensity, cold/drought stress, and nutrients deficiency stress (Jaakola, 2013; Su et al., 2014; Lotkowska et al., 2015; Xie et al., 2017). The accumulation of anthocyanin is often considered to be associated with stress defense (Xu et al., 2017).

Hydrogen gas (H_2_) is a colorless and odorless inert gas. Ohsawa et al. (2007) found that H_2_ could be used as a potential medical gas to selectively scavenge intracellular hydroxyl radicals and peroxynitrite in rats. Furthermore, H_2_ aqueous solution—hydrogen-rich water (HRW) has been used in the clinically treatment of colon inflammation, ischemic brain injury, and diabetes, etc. (Kajiya et al., 2009; Kamimura et al., 2011; Han et al., 2015). Studies in plants also showed that H_2_ has a number of biological effects. For instance, H_2_ has been reported to alleviate paraquat-induced oxidant stress in alfalfa, high salt stress in *Arabidopsis*, cadmium stress in Chinese cabbage and high light intensity stress in maize (Xie et al., 2012; Jin et al., 2013; Wu et al., 2015; Zhang et al., 2015). Previous studies also reported that H_2_ could regulate cucumber adventitious root formation (Zhu et al., 2016) and prolong the shelf life in kiwifruit (Hu et al., 2018). In our previous study, we found that the application of HRW under white light, which is the normal growth condition for radish sprouts, dose not stimulate anthocyanin accumulation. However, the application of HRW under UV-A could significantly increase anthocyanin accumulation in radish sprouts hypocotyls (Su et al., 2014). However, the underlying molecular mechanisms, especially the downstream signal molecules involved in HRW-regulated anthocyanin accumulation under UV-A remain to be elucidated.

Calcium (Ca^2+^) is an essential nutrient element for plant growth and development. In addition, Ca^2+^ is a ubiquitous secondary-messenger molecule, which participates in the signal transduction in plant development and plant resistance to both biotic and abiotic stresses (Kader and Lindberg, 2010; Schulz et al., 2013). Furthermore, it has been reported that the expression of *DFR* gene, which is a key gene involved in anthocyanin biosynthesis, was induced by Ca^2+^ (Gollop et al., 2002). Several studies have also demonstrated that Ca^2+^ stimulates the accumulation of anthocyanin (Li et al., 2004; Shin et al., 2013; Xu et al., 2014). The 1,4,5-trisphosphate (IP3) receptors (IP3Rs), located in endoplasmic reticulum membrane or tonoplast, play key roles in the regulation of Ca^2+^ signals. Binding of IP3 to the IP3Rs causes the calcium channel to open, and thus calcium ions could flow from the calcium stores to the cytoplasm (Handy et al., 2017). IP3 was shown to mediate ABA-induced synthesis of isoflavones in soybean sprouts under UV-B irradiation (Jiao et al., 2016). Studies have also shown that CaM can regulate the accumulation of anthocyanins in *Daucus carota* and grape fruits (Sudha and Ravishankar, 2003; Peng et al., 2016). The activity of CaM in the *Alternanthera bettzickiana* L. seedlings has a positive correlation with anthocyanin content (Wang et al., 2005). However, the function of Ca^2+^ in HRW-regulated anabolism of anthocyanin in radish sprouts exposed to UV-A is still unknown.

In this study, we observed an increment in cytoplasm Ca^2+^ concentration in radish protoplast when HRW was applied, which mimicked the effects of CaCl_2_. Pharmacological and molecular experiments suggested that Ca^2+^ could act as a downstream signal molecule in HRW-promoted anthocyanin accumulation under UV-A. These results are expected to further elucidate the mechanism of HRW-regulated anthocyanin biosynthesis. Furthermore, this study could also extend our knowledge of functional mechanism of H_2_ in higher plants and provide a theoretical basis and guidance for the production of anthocyanin-rich radish sprouts in greenhouse and environment-controlled facilities.

## Materials and methods

### Plant materials and growth conditions

Radish (*Raphanus sativus* L.) seeds were sterilized by 0.7% NaClO and soaked in deionized water at 25°C for 6 h, and then uniform seeds were selected and spread evenly in growth trays and cultured in a dark incubator at 25°C. After incubation for 36 h, sprouts were transferred into an environment-controlled chamber and exposed to UV-A irradiation (central wavelength 365 nm; the employed UV-A intensity was 5.5 W m^−2^). At the same time, sprouts were incubated in different treatment solutions as described in the corresponding figure legends. The sample without chemicals was the control (Con). The treatment solutions were replaced every 12 h with new prepared solution. The relative humidity and temperature was 80% and 25°C, respectively. After various treatments, sprouts were photographed and hypocotyl tissues were used immediately or frozen with liquid nitrogen and stored at −80°C for further use.

### The preparation of HRW

Purified H_2_ gas (99.99%, v/v) generated from a hydrogen gas generator (SHC-300, Saikesaisi Hydrogen Energy Co., Ltd, Jinan, China) was bubbled into 1,000 mL distilled water (pH 5.87, 25°C) at a rate of 150 mL min^−1^ for 60 min. In our experimental conditions, this is a sufficient time to saturate the solution with H_2_. The saturation of H_2_ in water was 781 μmol L^−1^ at 25°C. The H_2_ concentration in freshly prepared HRW (100% saturation) analyzed by a needle-type Hydrogen Sensor (Unisense) was 830 μmol L^−1^. After 12 h, the concentration of H_2_ in HRW still maintained about 150 μmol L^−1^, which was much higher than 0.3 μmol L^−1^ in sprouts in normal condition (see Figure S1).

### Measurement of anthocyanin content

Anthocyanin content was determined according to the mothed of Xie et al. (2016) with slight modifications. Briefly, 0.5 g of hypocotyl samples were soaked in 5 mL 1% HCl-methanol solution and incubated for 12 h at 4°C. The absorbancies of the extracts at 530 and 675 nm were measured by spectrophotometical meter. And (A_530_-0.25 × A_657_) per gram fresh weight was used to quantify the relative amount of anthocyanin.

### Measurement of cytosolic calcium concentration

The radish mesophyll protoplasts were isolated according to the methods of Yoo et al. (2007) and Hagimori and Nagaoka (1992) with minor modifications. 0.5 mm leaf strips were cut from the middle part of the leaves and transferred to the enzyme solution [0.5 M MES (pH 7.5) containing 1.5% (w/v) cellulase R10, 0.4% macerozyme R10, 0.4 mM mannitol, 20 mM KCl, 10 mM CaCl_2_, and 0.1% BSA] immediately. After vacuum infiltrated in the dark for 30 min at 25°C, the leaf strips were transferred to an incubator to continue the digestion in the dark for 4 h at room temperature. Then the enzyme solution was washed twice with W5 solution. The protoplasts were collected after centrifugation and re-suspended in MMG [4 mM 4-morpholineethanesulfonic acid (MES, pH 5.7) containing 0.4 M mannitol and 15 mM MgCl_2_] solution. Protoplasts were kept at room temperature.

Cytosolic calcium concentration was measured by the calcium fluorescent probe Fluo-3/AM (Molecular Probes) based on the method of Yan et al. (2015) and Zhang et al. (1998). Briefly, 1 mM Fluo-3/AM in anhydrous DMSO (5 μL) was added to radish mesophyll protoplasts. After incubation at 4°C in the dark for 2 h, the protoplasts were washed three times with isotonic solution to wash away the residual dye. Then the protoplasts were incubated with incubation solution [containing 20 mM Fluo-3/AM, 0.5 M mannitol, 4 mM MES (pH 5.7), and 20 mM KCl] at 25°C for 1 h. The fluorescence of Ca^2+^ was measured after various reagents (as described in **Figure 2**) were added. Images of 25 protoplasts for each treatment in three independent experiments were observed using a PE (Ultra View VOX) laser scanning confocal microscope (LSCM) with the 488 nm excitation wavelength. The emission fluorescence was filtered by a 515 nm filter to eliminate the autofluorescence of chlorophyll. The fluorescence of protoplasts on acquired images were analyzed by image J software (NIH). Data were calculated as means ± SE of pixel intensities.

### Anthocyanin profiles analyzed by ultra-performance liquid chromatography-mass spectrometric (UPLC-MS)

The qualitative analysis of anthocyanin profiles was based on our previous work (Su et al., 2014). For anthocyanin extraction, 2 g of fresh hypocotyl samples were homogenized with 6 mL 1% HCL-methanol, and then the suspensions were placed in an ultrasonic bath for 10 min at 20°C. After centrifugation, 5 mL supernatant was taken out, and the residues were extracted with 6 mL extract solution again. The supernatants were combined. Then, daidzein was added as an internal standard to a final concentration of 0.03 μg mL^−1^. After vacuum-drying, 500 μL of 80% methanol was added to dissolve the anthocyanin. And the supernatants were filtered through 0.22 μm membrane for further analysis.

The samples were analyzed by LC–MS system (G2-XS QTof, Waters). Two microliter solution was injected into the UPLC column (2.1 × 100 mm, ACQUITY UPLC BEH C18 column containing 1.7 μm particles) with a flow rate of 0.4 mL min^−1^. The mobile phase A consisted of 0.1% formic acid in water, and mobile phase B consisted of 0.1% formic acid in acetonitrile. The solvent was administered according to the following protocol: 5% of B in 2 min, from 5 to 95% of B in 15 min, 95% of B in 2 min, and from 95 to 5% of B in 5 min. Mass spectrometry was performed using electrospray source in positive ion mode with MSe acquisition mode, with a selected mass range of 50–1,200 *m/z*. The lock mass option was enabled using leucine-enkephalin (*m/z* 556.2771) for recalibration. The ionization parameters were the following: capillary voltage was 2.5 kV, collision energy was 40 eV, source temperature was 120°C, and desolvation gas temperature was 400°C. Data acquisition and processing were performed using Masslynx 4.1.

### Determination of IP3 content and CaM content

IP3 extraction was measured according to the method of Burnette et al. (2003). Briefly, 0.5 g fresh sample of hypocotyl tissues were ground to a fine powder in liquid nitrogen. 0.5 mL 20% perchloric acid (v/v) was added and the mixture was incubated on ice for 20 min. After centrifugation (4°C, 2,000 g, 15 min), the precipitated protein was removed. The neutralized supernatant fraction was used to assay the IP3 content using an IP3 content assay kit (GE Healthcare) according to the manufacturer's instructions.

For CaM extraction, 0.5 g fresh hypocotyl samples were ground to a fine powder in liquid nitrogen. Then 2.5 mL extraction solution (50 mM tris-HCl containing 1 mM EGTA, 0.5 mM PMSF, and 1 mM β-mercaptoethanol) was added, and the mixture was incubated on ice for 20 min. After centrifugation at 12,000 g for 30 min at 4°C, the supernatant was taken out and incubated at 90°C for 3 min and then incubated on the ice immediately. The extraction was centrifuged at 12,000 g for 30 min at 4°C and the supernatant was collected for the measurement of protein and CaM content. For CaM content assay, plant CaM content assay kit (Kmaels) was used.

### Determination of enzyme activities

For the analysis of enzyme activities, all operations were carried out at 4°C. The activity of anthocyanin-related enzymes in hypocotyl tissues referred to the enzyme activity in the crude protein fractions. PAL, CHS and CHI were extracted according to Li et al. (2013). For PAL and CHI extraction, 1 g of frozen radish hypocotyl tissues were ground to fine powder in liquid nitrogen and 3 mL of 100 mM Tris-HCl buffer (pH 8.8, containing 14 mM β-mercaptoethanol, 5 mM DTT, 1% BS and 5% PVPP) was added. The homogenate was centrifuged at 12,000 g for 20 min at 4°C. The supernatant was used for PAL and CHI enzyme activity assay according to the methods of Ren and Sun (2014) and Lister et al. (1996), respectively. For CHS extraction, 2 g frozen radish hypocotyl tissues were homogenized with 5 mL of 100 mM sodium phosphate buffer (pH 6.8, containing 5% PVPP, 14 mM β-mercaptoethanol, 5 mM DTT, 40 mM sodium ascorbate, 10 μM leupetin, 3 mM EDTA, and 2% BSA). The homogenate was centrifuged at 12,000 g for 20 min at 4°C. Then the supernatant was precipitated with ammonium sulfate [70% (w/v) saturation], kept on ice for 1 h, and centrifuged again. The resulting precipitate was dissolved in 1 mL of 100 mM sodium phosphate buffer (pH 6.8, containing 8 mM DTT, 40 mM sodium ascorbate, and 1% BSA). CHS activity was assayed using plant CHS activity assay kit (GE Healthcare) according to the manufacturer's instructions.

DFR crude protein were extracted according to the methods of Miyagawa et al. (2015). Briefly, 0.5 g frozen hypocotyl tissues were ground in liquid nitrogen and then suspended in 3 mL 0.1 M Tris-HCl buffer (pH 7.0), then centrifuged at 12,000 g for 15 min at 4°C. The proteins in the supernatant fraction were precipitated with 80% saturated ammonium sulfate and collected by centrifugation. The resulting precipitate was dissolved in 50 μL of extraction buffer. The DFR activity was assayed using plant DFR ELISA kit (Janelly, Shanghai, China) according to the manufacturer's instructions. UFGT was extracted according to the methods of Li et al. (2013). UFGT activity were measured according to the methods of Zhang et al. (2014). Briefly, 10 μL of enzyme extract was added to the reaction mixture, which contained 10 mM buffer (Hepes-KOH, pH 8.0), 250 mM MgCl_2_, 2 mM DTT, 9 mM UDP-galactose, and 0.3 mM cyanidin, to a final volume of 200 μL. The reaction mixture was incubated at 37°C for 10 min and terminated by the addition of 50 μL 35% trichloroacetic acid. The UFGT activity was determined by HPLC at 525 nm.

### Isolation of total RNA and qRT-PCR

Total RNA was extracted from radish hypocotyls using Trizol reagent (Invitrogen, USA). One microgram of aliquots were treated with RNase-free DNase I to remove genomic DNA and then reverse transcribed using a RevertAid First Strand cDNA Synthesis Kit (Thermo Scientific, USA) according to the manufacturer's recommendation. qPCR was carried out on a Mastercycler ep realplex Real-time PCR System (Eppendorf, Hamburg, Germany) using Bestar SYBR Green qPCR Mastermix (DBI, Bioscience Inc., Germany) according to our previous methods (Wu et al., 2015). Reactions were performed at 95°C for 2 min, 40 cycles of 95°C for 10 s, 60°C for 30 s, and 72°C for 30 s. The specific primers were designed according to the reference unigene sequence using the Primer Premier 6.0 software (see Table S1 in the Supplementary Material). *Actin2* and *EF1* were used as the reference genes (Xu et al., 2012). The relative expression levels of the selected genes were calculated using the 2^−ΔΔCT^ approach, with normalization of data to the geometric average of two reference control genes (Vandesompele et al., 2002).

### Statistical analysis

Results were expressed as the means ± SE (standard error) of at least three independent experiments. All the data were subjected to one-way ANOVA analysis (SPSS Statistics 17.0 software). Duncan's multiple range test was carried out to determine significant differences at *P* < 0.05.

## Results

### Anthocyanin content was induced by HRW and CaCl_2_ under UV-A irradiation

**Figure 1:**
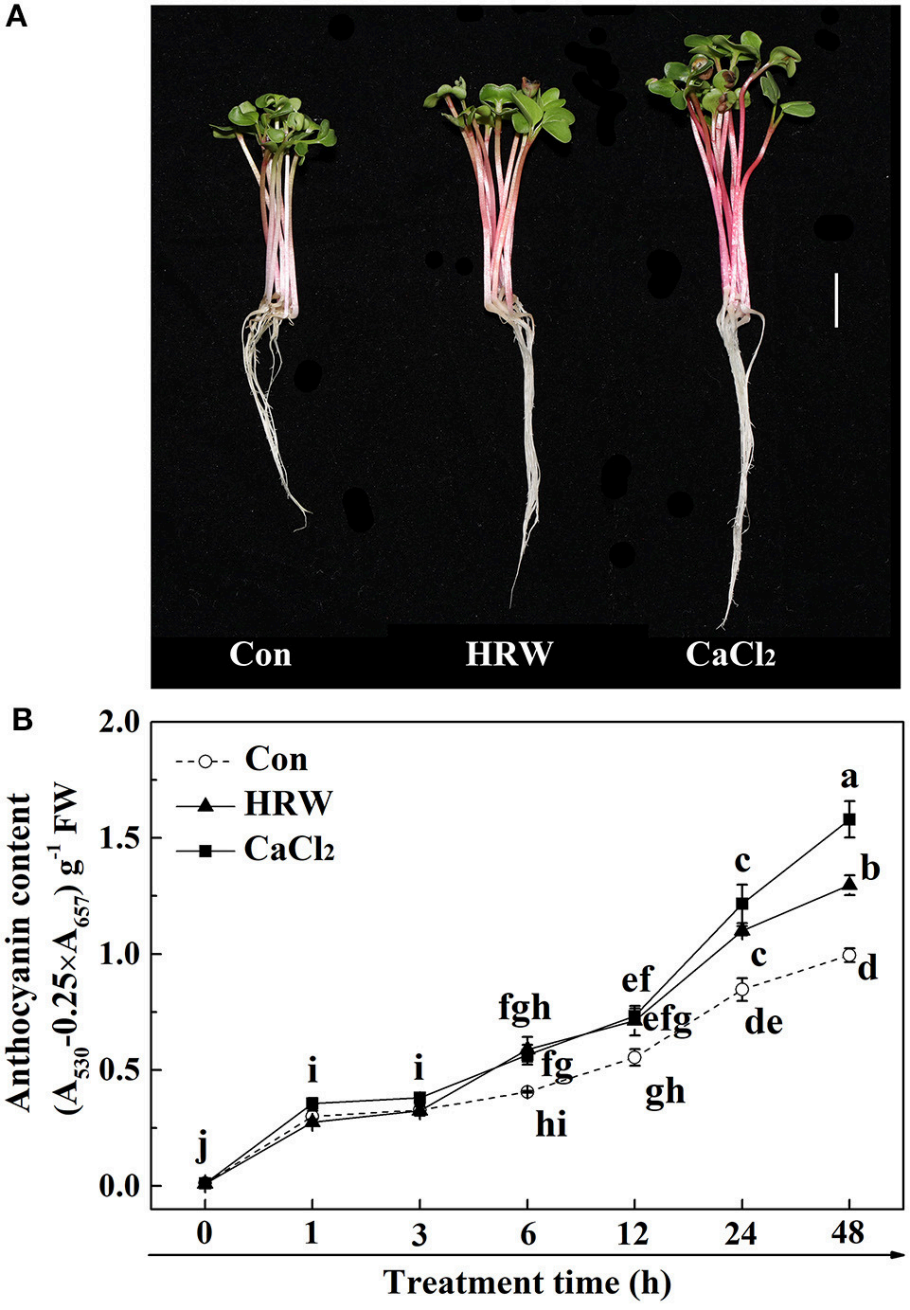
Effects of HRW and CaCl_2_ on morphology of radish sprouts **(A)**, bar = 1 cm, and anthocyanin content of radish sprouts hypocotyls **(B)** under UV-A irradiation. After cultured in distilled water in the dark for 36 h, radish sprouts were incubated in solution containing HRW (100% saturation) or 10 mM CaCl_2_ under UV-A irradiation. The sample with distilled water alone was the control (Con). The photographs were taken after 48 h plant growth under UV-A **(A)**. Anthocyanin content was measured during plant growth at 0, 1, 3, 6, 12, 24 and 48 h after exposed to UV-A **(B)**. Values are means ± SE (*n* = 3). Measurement in the same treatment time followed by different letters are significantly different at *P* < 0.05 according to Duncan's multiple test.

The effects of HRW and CaCl_2_ on anthocyanin accumulation under UV-A irradiation were shown in Figure 1. In the control condition, the anthocyanin content in radish sprouts increased gradually during the culture time. In comparison to the control, both HRW and CaCl_2_ treatments accelerated the increase of anthocyanin accumulation. Among them, the content of anthocyanin under CaCl_2_ treatment at 12 h was slightly higher than that of the control. In addition, the differences in anthocyanin content between the two treatment groups and the control group increased as treatment time increased. At 48 h, the content of anthocyanin under HRW and CaCl_2_ treatments were significantly higher than that of the control, and the content of anthocyanin under CaCl_2_ treatment was the highest, significantly higher than that of other treatments.

### HRW promoted the concentration of cytosolic calcium

**Figure 2:**
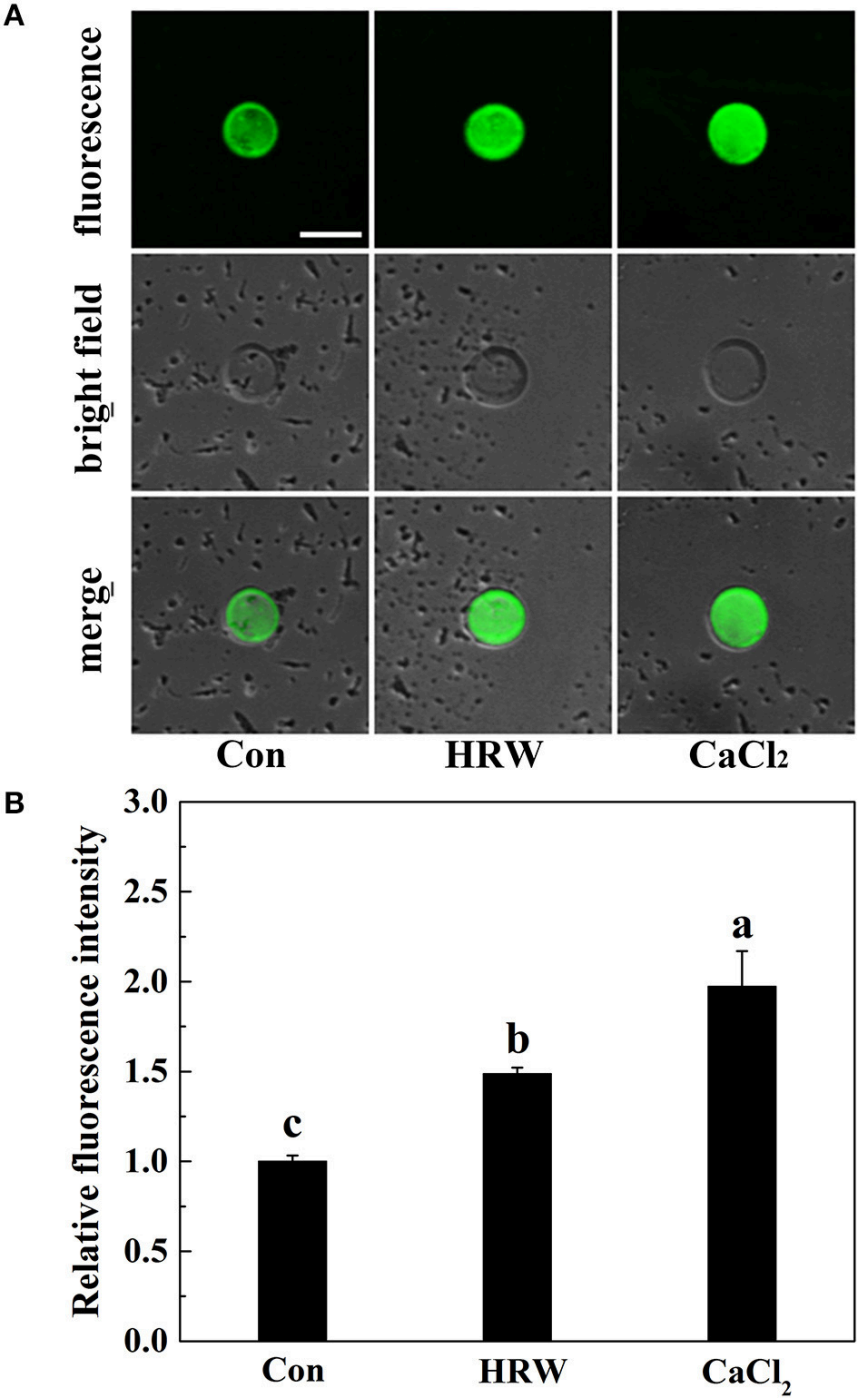
Changes of cytosolic calcium concentration in protoplasts of radish mesophyll cells. Radish mesophyll protoplasts were loaded with Fluo-3/AM, and then incubated in an isotonic solution containing 10 mM CaCl_2_ or HRW (100% saturation) for 2 h under UV-A irradiation. Afterwards, the fluorescence of Fluo-3/AM was detected by LSCM **(A)**. Bar = 50 μm. The typical fluorescence images **(A)** and corresponding fluorescence intensities **(B)** were given. The sample with isotonic solution alone was the control (Con). The fluorescence intensities are mean ± SE (*n* = 3). Bars with different letters are significantly different in comparison with Con at *P* < 0.05 according to Duncan's multiple test.

To investigate the possible role of cytosolic calcium in HRW-regulated anthocyanin accumulation in radish sprouts, we first examined the cytosolic calcium concentration. Radish mesophyll protoplasts treated with different reagents were loaded with calcium fluorescent probe Fluo-3/AM. The protoplasts of the control samples showed the basal cytosolic calcium concentration (Figure 2). Similar to the treatment of CaCl_2_, HRW resulted in a significant increase in the cytosolic calcium concentration compared to control treatment. These results suggested that calcium may be one of the important signaling components in HRW-triggered accumulation of anthocyanin under UV-A in radish sprouts.

### HRW-induced anthocyanin accumulation was blocked by the specific calcium antagonists

Based on our hypothesis, calcium antagonists were exogenously applied. Compared with the control (UV-A treatment group), CaCl_2_ addition significantly increased the anthocyanin content, while the calcium ion chelator EGTA, IP3 biosynthesis inhibitor neomycin significantly reduced the anthocyanin content (Figure S2, Table 1). Whereas, calcium antagonists LaCl_3_ and RR had no effects on anthocyanin content (Figure S2). These results indicated that IP3-mediated calcium signal might take part in HRW-regulated anthocyanin biosynthesis. In the subsequent experiment, EGTA and neomycin were chosen as calcium antagonists.

**Table 1:** Anthocyanin profile of radish sprouts hypocotyls with different treatments under UV-A.

**No**.	**Compound**	**Treatment**							
		**Con**	**Con + CaCl_2_**	**Con + EGTA**	**Con + Neomycin**	**HRW**	**HRW + CaCl_2_**	**HRW + EGTA**	**HRW + Neomycin**
1	Cyanidin-3,5-*O*-diglucoside	0.3599 ± 0.0586c	0.1608 ± 0.0150d	0.1418 ± 0.0142d	0.1174 ± 0.0100d	0.3695 ± 0.0037c	1.0286 ± 0.0015a	0.6169 ± 0.0296b	0.3561 ± 0.0115c
2	Cyanidin-3-*O*-(6-*O*-(E)-4-coumaroyl-β-D-glucoside)	ND	ND	ND	ND	0.1909 ± 0.0031b	0.5550 ± 0.0248a	0.1633 ± 0.0048c	0.1378 ± 0.0031c
3	Cyanidin-3-*O*-(6-*O*-para-coumaroyl)glucoside-5-*O*-glucoside	ND	ND	ND	ND	0.1006 ± 0.0002b	0.1616 ± 0.0020a	ND	ND
4	Cyanidin-3-*O*-rutinoside 5-*O*-β-D-glucoside	2.8541 ± 0.0547d	10.0009 ± 0.0001b	2.3452 ± 0.1076ef	1.7116 ± 0.1245f	6.3203 ± 0.2363c	12.7610 ± 0.3597a	5.8785 ± 0.3455c	6.4453 ± 0.3606c
5	Cyanidin-3-*O*-rutinoside	5.9632 ± 0.1117e	21.3833 ± 0.0274b	3.3792 ± 0.1539f	4.1655 ± 0.3154f	15.5159 ± 0.2914c	23.2378 ± 0.1098a	14.2688 ± 0.3192d	6.4289 ± 0.7841e
6	Delphinidin-3-*O*-(6-*O*-(E)-4-coumaroyl-β-D-glucoside)	ND	0.2209 ± 0.0245a	ND	ND	0.1925 ± 0.0029a	0.2033 ± 0.0081a	0.1137 ± 0.0038b	ND
7	Delphinidin-3-*O*-arabinoside	0.2348 ± 0.0102c	0.3180 ± 0.0137b	0.2241 ± 0.0048c	0.1806 ± 0.0273d	0.3669 ± 0.0114a	0.3877 ± 0.0063a	0.2358 ± 0.0143c	0.1391 ± 0.0135d
8	Delphinidin-3-*O*-rutinoside	ND	0.1742 ± 0.0213d	ND	ND	0.1383 ± 0.0142e	1.0319 ± 0.0029a	0.6409 ± 0.0093b	0.3600 ± 0.0097c
9	Delphinidin-3-*O*-rhamnoside chloride	ND	ND	ND	ND	0.1340 ± 0.0012a	0.1365 ± 0.0021a	ND	ND
10	Pelargonidin-3-*O*-glucoside	0.2690 ± 0.0183d	1.2818 ± 0.1164b	0.1511 ± 0.0038d	0.1563 ± 0.0270d	1.4600 ± 0.0234b	2.4378 ± 0.154a	1.0307 ± 0.0086c	2.2313 ± 0.1189a
11	Petunidin-3-*O*-galactoside	0.0393 ± 0.0107d	0.2273 ± 0.0074a	0.2303 ± 0.0106a	0.1299 ± 0.0146b	0.1060 ± 0.0073bc	0.1236 ± 0.0072bc	0.1000 ± 0.0000c	ND
Total anthocyanin		9.7202 ± 0.2198f	33.7672 ± 0.1034b	6.4384 ± 0.1613g	6.4614 ± 0.4336g	24.8948 ± 0.5535c	42.0647 ± 0.3134a	23.0488 ± 0.7105d	16.0984 ± 0.8664e

Data are presented as means ± SE (n = 3).

Values followed by different letters are significantly different according to Duncan's multiple test at P < 0.05.

ND, Not Detectable.

### The profile analysis of anthocyanin

Based on our previous experiments, the major anthocyanin components were determined by using UPLC-MS to evaluate the effects of CaCl_2_, EGTA, and neomycin on HRW-promoted anthocyanin biosynthesis under UV-A. In this study, a total of 11 anthocyanins were detected in radish sprouts hypocotyls (Table 1), including cyanidin, delphinidin, pelargonidin and petunidin's derivatives. Among them, Cyanidin-3-*O*-rutinoside-5-*O*-β-D-glucoside and Cyanidin-3-*O*-rutinoside (two cyanidin derivatives) exhibited the highest abundance in radish sprouts hypocotyls, followed by Cyanidin-3,5-*O*-diglucoside and Pelargonidin-3-*O*-glucoside. In the absence of HRW, CaCl_2_ increased the anthocyanin components from 6 to 8. Compared with the control, CaCl_2_ significantly increased the content of most of the anthocyanin components. For example, the content of Cyaniding-3-*O*-rutinoside-5-*O*-β-D-glucoside and Cyanidin-3-*O*-rutinoside under CaCl_2_ treatment was 3.50- and 3.59-fold higher than the control, respectively. Whereas EGTA and neomycin significantly decreased both the components and content of anthocyanins. In comparison to the control, HRW increased the components of anthocyanins to 11 and significantly increased the content of Cyanidin-3,5-*O*-diglucoside, Cyanidin-3-*O*-rutinoside and Pelargonidin-3-*O*-glucoside. It is also important to point out that the amount of the above three anthocyanins were further increased by HRW + CaCl_2_ treatment. However, the co-treatment of HRW with EGTA or neomycin decreased the number of anthocyanins and significantly inhibited anthocyanin contents. Besides, some anthocyanin monomer contents were significantly decreased by EGTA and neomycin, whether HRW was presence or not. For instance, in the absence of HRW, the content of cyanidin-3-*O*-rutinoside upon EGTA and neomycin treatment accounted for 62 and 57% of that of the control, respectively. While in the presence of HRW, the content of cyanidin-3-*O*-rutinoside upon EGTA and neomycin treatment accounted for 92 and 41% of that of HRW treatment, respectively.

Further analysis showed that the total anthocyanin content was significantly increased upon CaCl_2_ treatment (3.47-fold than the control), but significantly decreased upon EGTA and neomycin treatment (decreased by 33.76 and 33.52% than the control, respectively). It was noteworthy that the total anthocyanin content was significantly increased when treated with HRW (2.56-fold than the control) and was further significantly increased when treated with HRW + CaCl_2_ (4.33-flod than the control). However, when treated with HRW + EGTA and HRW + Neomycin, total anthocyanin content significantly decreased than the HRW treatment. Thus, these results revealed that both the component and content of anthocyanin in radish sprouts were affected by CaCl_2_ and calcium antagonists (EGTA and neomycin). It further suggested that IP3-mediated calcium signal plays an important role in HRW-regulated anthocyanin biosynthesis under UV-A.

### Effects of CaCl_2_ and calcium antagonists on IP3 content

**Figure 3:**
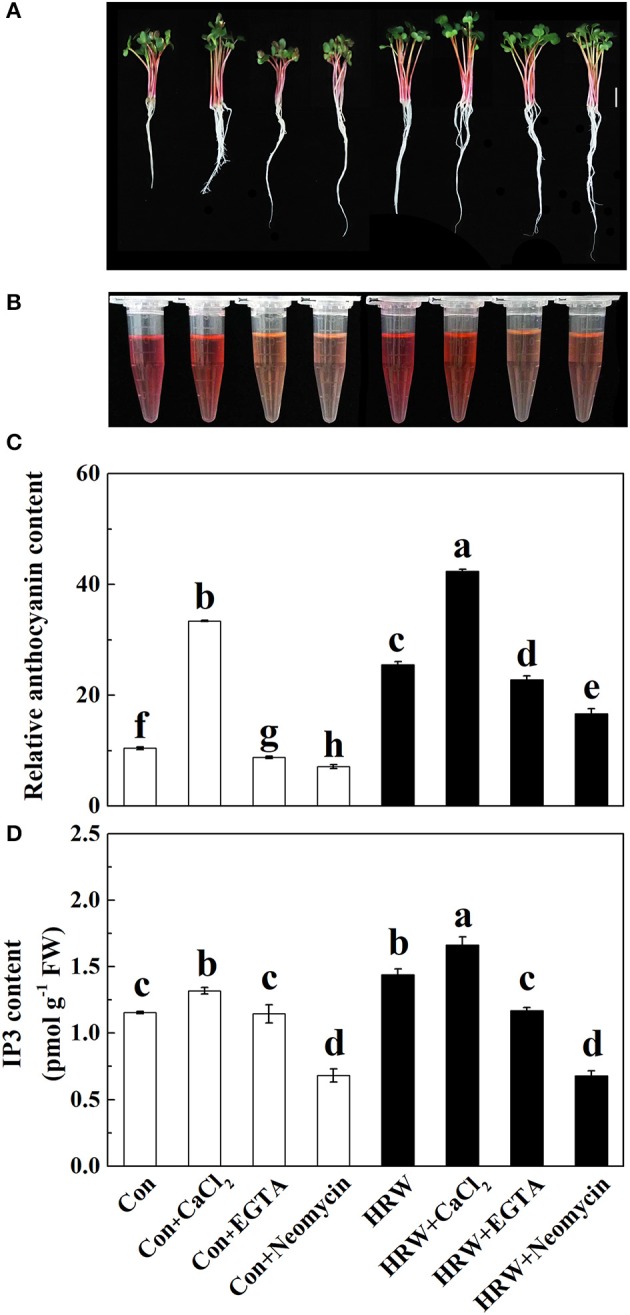
Effects of HRW, CaCl_2_ and Ca^2+^ antagonists on total anthocyanin content **(A–C)** and IP3 content **(D)** of radish sprouts hypocotyls under UV-A irradiation. After cultured in distilled water in the dark for 36 h, radish sprouts were incubated in distilled water or HRW (100% saturation) containing 10 mM CaCl_2_, 1 mM EGTA or 1 mM Neomycin under UV-A. The sample with distilled water alone was the control (Con). The hypocotyl color **(A)**, anthocyanin extraction color **(B)** and total anthocyanin content **(C)** were measured at 48 h after exposed to UV-A. The IP3 content was measured at 24 h after exposed to UV-A **(D)**. Data are presented as means ± SE (*n* = 3). Bars with different letters are significantly different at *P* < 0.05 according to Duncan's multiple test.

To confirm the role of IP3 in HRW-promoted anthocyanin biosynthesis, IP3 content was measured. Compared with the control, IP3 content was significantly increased with CaCl_2_, but significantly decreased with neomycin treatment (Figures 3A–C). In the presence of HRW, the same phenomenon was observed. We also noticed that the incubation of radish sprouts with EGTA, whether HRW was present or not, had no effects on IP3 content (Figure 3C). However, EGTA decreased total anthocyanin content in HRW-treated samples (Figures 3B,C). Overall, the trend of IP3 content in all treatments coincided with that of total anthocyanin content (Figure 3). These results indicated that IP3-dependent calcium signaling pathway might be involved in HRW-regulated anthocyanin biosynthesis under UV-A.

### Activation of anthocyanin biosynthetic enzymes

**Figure 4:**
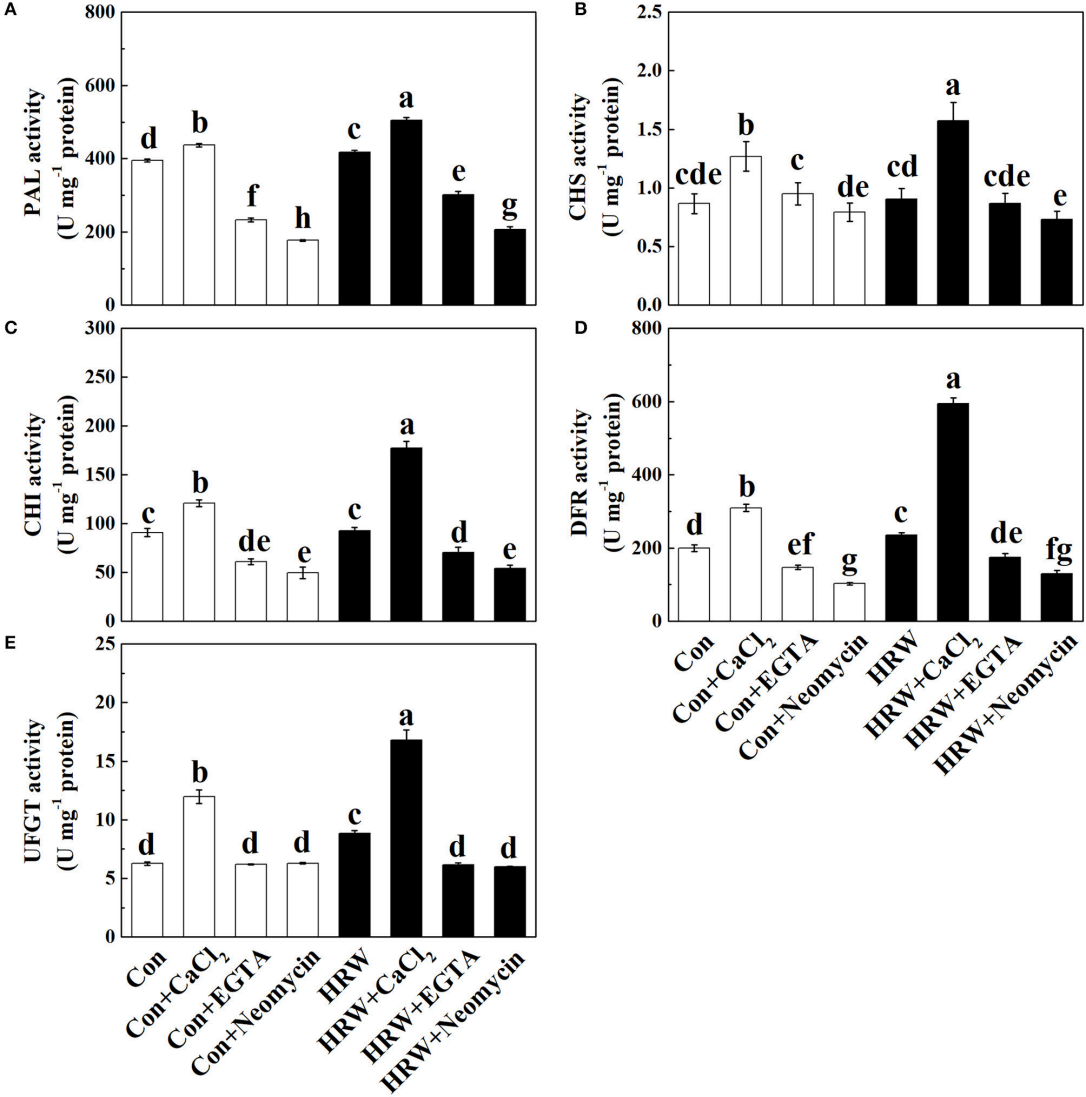
Effects of HRW, CaCl_2_ and Ca^2+^ antagonists on activity of anthocyanin biosynthesis-related enzymes in radish sprouts hypocotyls under UV-A irradiation. Radish sprouts were further incubated in distilled water or HRW (100% saturation) containing 10 mM CaCl_2_, 1 mM EGTA or 1 mM Neomycin under UV-A irradiation. The sample with distilled water alone was the control (Con). The activity of PAL **(A)**, CHS **(B)**, CHI **(C)**, DFR **(D)** and UFGT **(E)** were measured at 24 h plant growth under UV-A. Data are presented as means ± SE (*n* = 3). Bars with different letters are significantly different at *P* < 0.05 according to Duncan's multiple test.

The analysis of anthocyanin biosynthetic enzymes showed that CaCl_2_ significantly increased the activity of PAL, CHS, CHI, and DFR in the crude protein extractions prepared from the hypocotyl tissues, whereas EGTA and neomycin significantly decreased the activity of PAL, CHI, and DFR, compared with the control (Figures 4A–D). HRW significantly increased the activity of PAL and DFR, which was further increased by HRW + CaCl_2_ (Figures 4A,D). Meanwhile, HRW-induced increase of PAL, CHI and DFR activity was decreased by EGTA and neomycin (Figures 4A,C,D). Besides, HRW-induced CHS activity was significantly decreased by neomycin (Figure 4B). For the activity of UFGT, CaCl_2_, and HRW significantly increased UFGT activity. Meanwhile, compared to HRW treatment, HRW + CaCl_2_ further increased UFGT activity. However, EGTA and neomycin significantly decreased UFGT activity only in the presence of HRW.

### Transcript levels of anthocyanin biosynthetic-related genes

**Figure 5:**
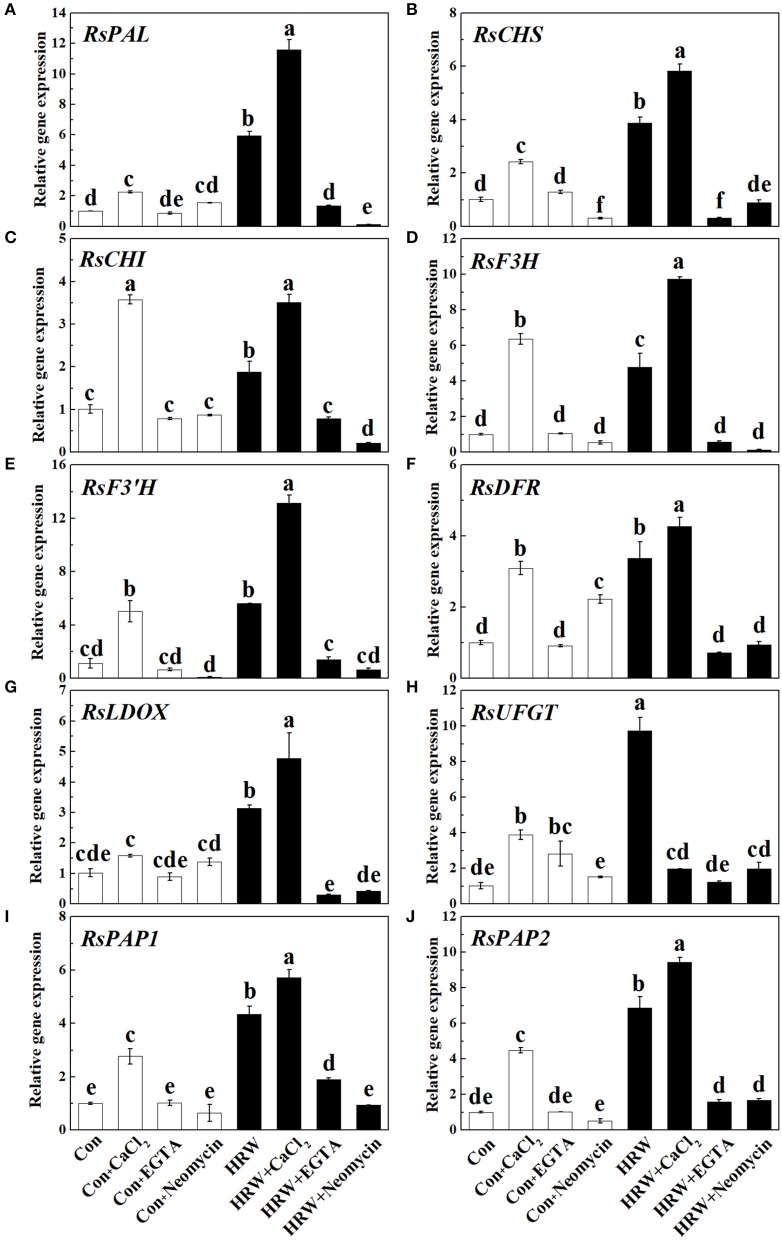
Expression of genes related to anthocyanin biosynthesis in radish sprouts hypocotyls under UV-A irradiation. Radish sprouts were further incubated in distilled water or HRW (100% saturation) containing 10 mM CaCl_2_, 1 mM EGTA or 1 mM Neomycin. The sample with distilled water alone was the control (Con). The gene expression levels of *RsPAL* **(A)**, *RsCHS* **(B)**, *RsCHI* **(C)**, *RsF3H* **(D)**, *RsF3'H* **(E)**, *RsDFR* **(F)**, *RsLDOX* **(G)**, *RsUFGT* **(H)**, *RsPAP1* **(I)** and *RsPAP2* **(J)** were analyzed by qRT-PCR at 12 h plant growth under UV-A. Data are presented as means ± SE (*n* = 3). Bars with different letters are significantly different at *P* < 0.05 according to Duncan's multiple test.

To further provide the molecular basis for above anthocyanin profile changes, the transcript levels of genes responsible for anthocyanin biosynthesis were analyzed by qRT-PCR. As shown in Figure 5, CaCl_2_ caused an increase in the expression level of all the genes detected (except for *RsLDOX*). These increasing tendencies were reversed by EGTA and neomycin. It is also important to note that the expression of two MYB transcription factors *RsPAP1* and *RsPAP2* was significantly promoted by HRW, and further promoted by HRW + CaCl_2_ (Figures 5I,J). Compared with the control, HRW significantly increased the expression of all the key genes. Furthermore, theses promoting effects were substantially strengthened by CaCl_2_ upon HRW treatment (except for *RsUFGT*). However, the expression levels of these key genes were down-regulated when EGTA or neomycin was co-treated with HRW. In general, the transcript levels of these key genes were in accordance with the changes of total anthocyanin content in radish sprouts hypocotyls.

### The possible involvement of CaM in HRW-regulated anthocyanin biosynthisis

**Figure 6:**
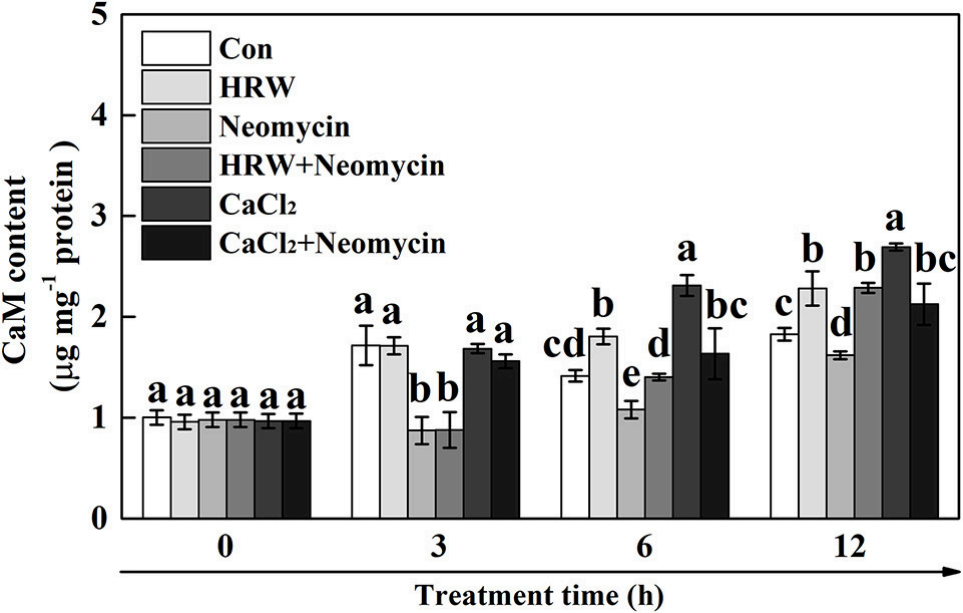
Effects of HRW, CaCl_2_, and Ca^2+^ antagonists on CaM content of radish sprouts hypocotyls under UV-A irradiation. After cultured in distilled water in the dark for 36 h, radish sprouts were incubated in HRW (100% saturation), 10 mM CaCl_2_, 1 mM EGTA or 1 mM Neomycin alone, or the combinations. The sample with distilled water alone was the control (Con). The CaM content was measured at 0, 3, 6 and 12 h plant growth under UV-A, respectively. Data are presented as means ± SE (*n* = 3). Bars with different letters are significantly different at *P* < 0.05 according to Duncan's multiple test.

To determine the role of CaM in HRW-regulated anthocyanin synthesis under UV-A, the dynamic changes of CaM abundance were measured (Figure 6). The results showed that the content of CaM significantly increased at 6 and 12 h incubation in HRW compared with the control, but significantly decreased when neomycin was co-treated. Similarly, CaM content significantly increased after 6 and 12 h incubation in CaCl_2_, and significantly decreased when neomycin was co-treated. Meantime, CaM content significantly decreased after incubation with Neomycin at 3, 6, and 12 h. To further clarify the involvement of CaM in HRW-regulated anthocyanin accumulation under UV-A, CaM inhibitor W7, W5, and TFP were exogenously added. As shown in Figure S3, CaM inhibitors W7 and TFP significantly reduced anthocyanin content. However, W5, a structural analog of W7, had no any effect on anthocyanin content.

## Discussion

Hydrogen gas was once considered as an insert gas that has no physiological effect on human body or plants. However, increasing number of studies in both mammalian and plant system have changed our previous standard stereotypes (Ohsawa et al., 2007; Ohta, 2012; Jin et al., 2013; Su et al., 2014; Chen et al., 2017). Recently, Xie et al. (2015) found that HRW could partially alleviated UV-B-induced oxidative damage by the manipulation of (iso)flavonoids metabolism in alfalfa. Our previous study also showed that HRW could increase UV-A-induced anthocyanin biosynthesis in radish sprouts hypocotyls (Su et al., 2014). However, the underlying mechanisms of HRW-regulated anthocyanin biosynthesis need to be fully clarified.

The biosynthesis of anthocyanin is regulated by environmental and developmental signals (Jaakola, 2013). Ca^2+^, a universal second messenger, plays an important role in the transduction of growth and development signaling, environmental signaling and hormone signaling (Yang and Poovaiah, 2003; Hetherington and Brownlee, 2004; Batistič and Kudla, 2012). The importance of Ca^2+^ for improving or maintaining fruit quality has long been recognized (Oms-Oliu et al., 2010; Zhi et al., 2017). However, there are relatively few studies on the effects of Ca^2+^ on the metabolism of anthocyanins and flavonoids. In this study, we provided strong evidence to illustrate the role of Ca^2+^ in HRW-induced anthocyanin biosynthesis in radish sprouts exposed to UV-A irradiation.

Firstly, we observed that HRW significantly increased the anthocyanin content, which mimicked the inducing effect of CaCl_2_ on the anthocyanin content (Figure 1). This result was consistent with a previous study in strawberry (Xu et al., 2014), showing that Ca^2+^ enhanced anthocyanin accumulation. Meanwhile, similar to the effect of CaCl_2_, the cytosolic Ca^2+^ concentration was significantly increased by HRW (Figure 2). Our results suggested that Ca^2+^ plays an important role in HRW-induced anthocyanin biosynthesis.

Secondly, the pharmacological experiments showed that Ca^2+^ chelator EGTA and IP3 synthesis inhibitor neomycin significantly inhibited the HRW-induced anthocyanin accumulation under UV-A (Figure S2, Table 1). These results were consistent with recent reports (Shin et al., 2013; Jiao et al., 2016), showing that EGTA and neomycin decreased anthocyanin content in *Arabidopsis* and soybean sprouts. It has been reported that soybean sprouts produced by soaking Ca^2+^ and spraying Ca^2+^ contained more isoflavone (Wang et al., 2016). The present study showed that Ca^2+^ and calcium antagonists affect both the composition and content of anthocyanins (Table 1). To an extent, the content of various anthocyanin monomers showed a similar trend under different treatments, that is the anthocyanin monomers content increased under CaCl_2_ treatment and decreased under EGTA and neomycin treatments, whether in the presence of HRW or not. Similar inhibition of isoflavone production was previously reported in soybean sprouts, showing that UV-B-induced isoflavone production in soybean sprouts was hindered by neomycin (Jiao et al., 2016). It was also noted that the total anthocyanin content increased significantly under CaCl_2_ treatment but decreased significantly under EGTA and neomycin treatments in the presence of HRW (Table 1). Meanwhile, the IP3 content under HRW treatment was significantly higher than that of the control and was significantly decreased under neomycin treatment (Figure 3), implying that HRW induced IP3 accumulation in radish sprouts hypocotyls. The above results indicated that Ca^2+^ signaling participates in the regulation of anthocyanin biosynthesis triggered by HRW, of which IP3-sensitive Ca^2+^ channels play a major role in our experimental conditions.

Thirdly, previous studies revealed that PAL, CHS, and CHI are the important early key genes in anthocyanin biosynthesis (Holton and Cornish, 1995; Jez et al., 2000; Rohde et al., 2004; Li et al., 2010), and DFR and UFGT are two important enzymes that catalyze the late steps of anthocyanin biosynthesis (Gonzalez et al., 2008). In the present study, the activity tendencies of the above mentioned key enzymes showed similar tendency of anthocyanin content (Figures 3, 4). Also, it has been suggested that *CHS, CHI, F3H*, and *F3*′*H* are the early biosynthetic genes (EBGs) in the flavonoid biosynthesis pathway which involved in the production of common precursors. And the downstream late biosynthetic genes (LBGs), including *DFR, LDOX*, and *UFGT*, are positively regulated by MYB transcription factors (Jaakola, 2013; Xu et al., 2015). In this study, the molecular evidence showed that the upregulation of *RsDFR, RsLDOX, RsUFGT, RsPAP1*, and *RsPAP2* induced by HRW were promoted after treated with CaCl_2_ but reversed after EGTA and neomycin treatments (Figures 5F–J). It is noteworthy that the expression level of upregulated genes (i.e., *RsPAL, RsCHS, RsCHI, RsF3H*, and *RsF3*′*H*) showed a similar expression pattern to the content of anthocyanin (Figures 5, 3C). This result is contrary to previous studies showing that MBW complexes regulate LBGs but not the EBGs nor the *PAL* (Koes et al., 2005; Jaakola, 2013). It has been well documented that PAP family is required specifically for anthocyanin accumulation and provides specificity to the MBW complex (Borevitz et al., 2000; Maier et al., 2013). Overexpressing *PAP1* and *PAP2* could promote anthocyanin accumulation in *Arabidopsis* and tobacco by up-regulating the entire phenylpropanoid pathway (Gonzalez et al., 2008; Mitsunami et al., 2014). In this study, the higher expression of *PAP1* and *PAP2* in radish sprouts treated with exogenous application of Ca^2+^ could, at least partly, explain the positive effect of exogenous Ca^2+^ on anthocyanin accumulation.

CaM was involved in sugar-induced anthocyanin biosynthesis in *Vitis vinifera* cells (Vitrac et al., 2000). Furthermore, changes of CaM activity were directly correlated with anthocyanin accumulation under low temperature in *A. bettzickiana* (Wang et al., 2005). Another research showed that calcium/calmodulin specifically bound FvUGT1 to enhance anthocyanin accumulation in strawberry (Peng et al., 2016). In this study, the promotion effect of HRW on CaM content is partially inhibited by neomycin (Figure 6). Subsequent pharmacological experiments showed that CaM inhibitor W7 and TFP significantly decreased anthocyanin content (Figure S3). These results provided the evidence for the contribution of CaM in terms of promoting HRW-triggered anthocyanin biosynthesis. Our results indicated that CaM probably takes part in calcium signal transduction to regulate HRW-triggered anthocyanin biosynthesis under UV-A.

## Conclusion

**Figure 7:**
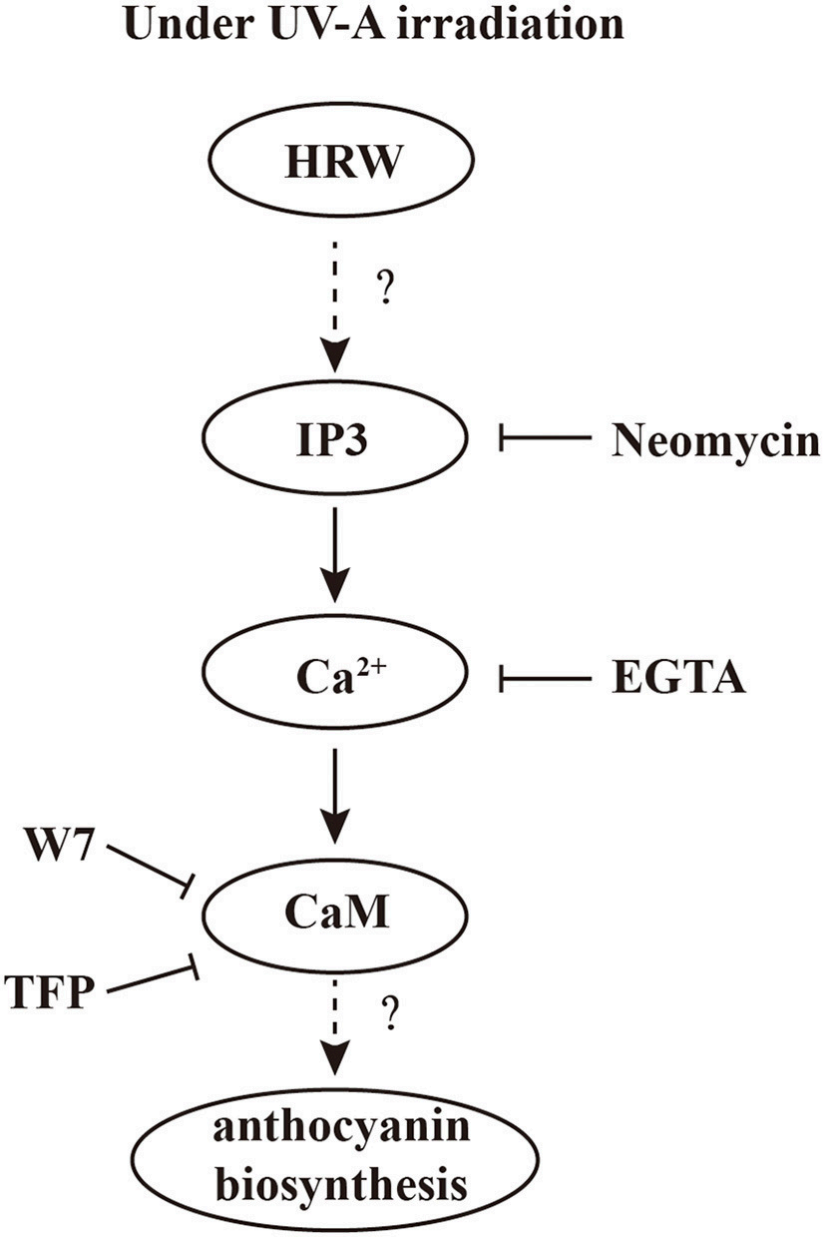
A proposed model of calcium involving in HRW-promoted anthocyanin biosynthesis under UV-A in radish sprouts hypocotyls. Under UV-A irradiation, HRW could promote the generation of IP3, which bound to the IP3 receptors of the calcium stores, and thus the concentration of cytosolic Ca^2+^ increased. Afterwards, calcium receptors CaM could activate the expression of anthocyanin biosynthesis-related genes to promote anthocyanin accumulation.

To our knowledge, using pharmacological and molecular approaches, which, in the case of multi-parameters, our data provide the first evidence that calcium signal plays an important role in HRW-triggered anthocyanin biosynthesis under UV-A (Figure 7). Under UV-A irradiation, HRW could promote the generation of IP3, which bind to the IP3 receptors of the calcium stores, and thus the concentration of cytosolic Ca^2+^ increased. Afterwards, calcium receptors CaM could activate the expression of anthocyanin biosynthesis-related genes to promote anthocyanin accumulation. In our study, the MYB transcription factors RsPAP1 and RsPAP2 played dominantly roles in this process. However, the interaction of calcium signaling and MBW complex should be further investigated. Moreover, the corresponding molecular evidence should be provided to uncover the targets of calcium signal, and then to well illustrate the regulation pattern of HRW-triggered anthocyanin biosynthesis under UV-A.

## Author contributions

JC and XZ conceived and designed the study. XZ and JW performed the experiments and analyzed the data. YH helped in performing the experiments. XC and WS provided the experimental methods. XZ wrote the manuscript, which was revised by NS and CL.

### Conflict of interest statement

The authors declare that the research was conducted in the absence of any commercial or financial relationships that could be construed as a potential conflict of interest.

## Abbreviations

EGTA: bis (β-aminoethylether)-N,N,N′N′-tetraacetic acid
IP3: inositol 1,4,5-trisphosphate
HRW: hydrogen-rich water
CaM: calmodulin
RR: ruthenium red
W7: N-(6-Aminohexyl)-5-chloro-1-naphthalenesulfonamid
W5: N-(6-Aminohexyl)-1-naphthalenesulfonamide Hydrochloride
TFP: trifluoperazine
Fluo-3/AM: Fluo-3 acetoxymethyl ester.

## References

[B1] Abdenour B. Charles R. (2014). Innovative anthocyanins formulation protects neuronal-like cells against oxidative stress-induced damage: pharmacotherapeutic application for Alzheimer's disease. Free Radic. Biol. Med. 75(Suppl. 1):S45. 10.1016/j.freeradbiomed.2014.10.804 26461383

[B2] Ali B. H. Cahliková L. Opletal L. Karaca T. Manoj P. Ramkumar A.. (2017). Effect of aqueous extract and anthocyanins of calyces of *Hibiscus sabdariffa* (Malvaceae) in rats with adenine-induced chronic kidney disease. J. Pharm. Pharmacol. 69, 1219–1229. 10.1111/jphp.12748 28542915

[B3] Batistič O. Kudla J. (2012). Analysis of calcium signaling pathways in plants. Biochim. Biophys. Acta. 1820, 1283–1293. 10.1016/j.bbagen.2011.10.012 22061997

[B4] Borevitz J. O. Xia Y. Blount J. Dixon R. A. Lamb C. (2000). Activation tagging identifies a conserved MYB regulator of phenylpropanoid biosynthesis. Plant Cell 12, 2383–2394. 10.1105/tpc.12.12.2383 11148285 PMC102225

[B5] Burnette R. N. Gunesekera B. M. Gillaspy G. E. (2003). An Arabidopsis inositol 5-phosphatase gain-of-function alters abscisic acid signaling. Plant Physiol. 132, 1011–1019. 10.1104/pp.019000 12805629 PMC167039

[B6] Chen Y. Wang M. Hu L. Liao W. B. Dawuda M. M. Li C. L. (2017). Carbon monoxide is involved in hydrogen gas-induced adventitious root development in cucumber under simulated drought stress. Front. Plant Sci. 8:128. 10.3389/fpls.2017.00128 28223992 PMC5293791

[B7] Glover B. J. Martin C. (2012). Anthocyanins. Curr. Biol. 22, R147–R150. 10.1016/j.cub.2012.01.021 22401890

[B8] Gollop R. Even S. Colova-Tsolova V. Perl A. (2002). Expression of the grape dihydroflavonol reductase gene and analysis of its promoter region. J Exp Bot. 53, 1397–1409. 10.1093/jexbot/53.373.1397 12021287

[B9] Gonzalez A. Zhao M. Leavitt J. M. Lloyd A. M. (2008). Regulation of the anthocyanin biosynthetic pathway by the TTG1/bHLH/Myb transcriptional complex in Arabidopsis seedlings. Plant J. 53, 814–827. 10.1111/j.1365-313X.2007.03373.x 18036197

[B10] Hagimori M. Nagaoka M. (1992). Nurse culture of Japanese radish (*Raphanus sativus* L.) mesophyll protoplasts. Plant Sci. 86, 105–113. 10.1016/0168-9452(92)90184-N

[B11] Han L. Tian R. Yan H. Pei L. Hou Z. Hao S.. (2015). Hydrogen-rich water protects against ischemic brain injury in rats by regulating calcium buffering proteins. Brain Res. 1615, 129–138. 10.1016/j.brainres.2015.04.038 25920370

[B12] Handy G. Taheri M. White J. A. Borisyuk A. (2017). Mathematical investigation of IP3-dependent calcium dynamics in astrocytes. J Comput. Neurosci. 42, 257–273. 10.1007/s10827-017-0640-1 28353176 PMC5756620

[B13] Harborne J. B. Williams C. A. (2000). Advances in flavonoid research since 1992. Phytochemistry 55, 481–504. 10.1016/S0031-9422(00)00235-1 11130659

[B14] Hetherington A. M. Brownlee C. (2004). The generation of Ca^2+^ signals in plants. Annu. Rev. Plant Biol. 55, 401–427. 10.1146/annurev.arplant.55.031903.141624 15377226

[B15] Holton T. A. Cornish E. C. (1995). Genetics and biochemistry of anthocyanin biosynthesis. Plant Cell 7, 1071–1083. 10.1105/tpc.7.7.1071 12242398 PMC160913

[B16] Hu H. L. Zhao S. P. Li P. X. Shen W. B. (2018). Hydrogen gas prolongs the shelf life of kiwifruit by decreasing ethylene biosynthesis. Postharvest Biol. Tec. 135, 123–130. 10.1016/j.postharvbio.2017.09.008

[B17] Jaakola L. (2013). New insights into the regulation of anthocyanin biosynthesis in fruits. Trends Plant Sci. 18, 477–483. 10.1016/j.tplants.2013.06.003 23870661

[B18] Jez J. M. Bowman M. E. Dixon R. A. Noel J. P. (2000). Structure and mechanism of the evolutionarily unique plant enzyme chalcone isomerase. Nat. Struct. Biol. 7, 786–791. 10.1038/79025 10966651

[B19] Jiao C. F. Yang R. Q. Gu Z. X. (2016). Cyclic ADP-ribose and IP3 mediate abscisic acid-induced isoflavone accumulation in soybean sprouts. Biochem. Biophys. Res. Commun. 479, 530–536. 10.1016/j.bbrc.2016.09.104 27664703

[B20] Jin Q. J. Zhu K. Cui W. Xie Y. Han B. Shen W. (2013). Hydrogen gas acts as a novel bioactive molecule in enhancing plant tolerance to paraquat-induced oxidative stress via the modulation of heme oxygenase-1 signalling system. Plant Cell Environ. 36, 956–969. 10.1111/pce.12029 23094798

[B21] Kader M. A. Lindberg S. (2010). Cytosolic calcium and pH signaling in plants under salinity stress. Plant Signal. Behav. 5, 233–238. 10.4161/psb.5.3.10740 20037468 PMC2881266

[B22] Kajiya M. Silva M. J. Sato K. Ouhara K. Kawai T. (2009). Hydrogen mediates suppression of colon inflammation induced by dextran sodium sulfate. Biochem. Biophys. Res. Commun. 386, 11–15. 10.1016/j.bbrc.2009.05.117 19486890

[B23] Kamimura N. Nishimaki K. Ohsawa I. Ohta S. (2011). Molecular hydrogen improves obesity and diabetes by inducing hepatic FGF21 and stimulating energy metabolism in *db/db* mice. Obesity 19, 1396–1403. 10.1038/oby.2011.6 21293445

[B24] Koes R. Verweij W. Quattrocchio F. (2005). Flavonoids: a colorful model for the regulation and evolution of biochemical pathways. Trends Plant Sci. 10, 236–242. 10.1016/j.tplants.2005.03.002 15882656

[B25] Kong J. M. Chia L. S. Goh N. K. Chia T. F. Brouillard R. (2003). Analysis and biological activities of anthocyanins. Phytochemistry 64, 923–933. 10.1016/S0031-9422(03)00438-2 14561507

[B26] Li P. Ma F. Cheng L. (2013). Primary and secondary metabolism in the sun-exposed peel and the shaded peel of apple fruit. Physiol. Plant. 148, 9–24. 10.1111/j.1399-3054.2012.01692.x 22989296

[B27] Li X. Bonawitz N. D. Weng J. K. Chapple C. (2010). The growth reduction associated with repressed lignin biosynthesis in *Arabidopsis thaliana* is independent of flavonoids. Plant Cell 22, 1620–1632. 10.1105/tpc.110.074161 20511296 PMC2899864

[B28] Li Z. H. Sugaya S. Gemma H. Iwahori S. (2004). The effect of calcium, nitrogen and phosphorus on anthocyanin synthesis in ‘Fuji’ apple callus. Acta Hortic. 653, 209–214. 10.17660/ActaHortic.2004.653.29

[B29] Lister C. E. Lancaster J. E. Walker J. R. L. (1996). Developmental changes in enzymes of flavonoid biosynthesis in the skins of red and green apple cultivars. J. Sci. Food Agric. 71, 313–320. 10.1002/(SICI)1097-0010(19990501)79:6<810::AID-JSFA288>3.0.CO;2-7

[B30] Lotkowska M. E. Tohge T. Fernie A. R. Xue G. P. Balazadeh S. Mueller-Roeber B. (2015). The arabidopsis transcription factor MYB112 promotes anthocyanin formation during salinity and under high light stress. Plant Physiol. 169, 1862–1880. 10.1104/pp.15.00605 26378103 PMC4634054

[B31] Maier A. Schrader A. Kokkelink L. Falke C. Welter B. Iniesto E.. (2013). Light and the E3 ubiquitin ligase COP1/SPA control the protein stability of the MYB transcription factors PAP1 and PAP2 involved in anthocyanin accumulation in Arabidopsis. Plant J. 74, 638–651. 10.1111/tpj.12153 23425305

[B32] Mitsunami T. Nishihara M. Galis I. Alamgir K. M. Hojo Y. Fujita K.. (2014). Overexpression of the PAP1 transcription factor reveals a complex regulation of flavonoid and phenylpropanoid metabolism in *Nicotiana tabacum* plants attacked by *Spodoptera litura*. PLoS ONE 9:e108849. 10.1371/journal.pone.0108849 25268129 PMC4182574

[B33] Miyagawa N. Miyahara T. Okamoto M. Hirose Y. Sakaguchi K. Hatano S. (2015). Dihydroflavonol 4-reductase activity is associated with the intensity of flower colors in delphinium. Plant Biotechnol. 32, 4445–4452. 10.5511/plantbiotechnology.15.0702b

[B34] Nam S. H. Choi S. P. Kang M. Y. Kozukue N. Friedman M. (2005). Antioxidative, antimutagenic, and anticarcinogenic activities of rice bran extracts in chemical and cell assays. J. Agric. Food Chem. 53, 816–822. 10.1021/jf0490293 15686439

[B35] Nijveldt R. J. van Nood E. van Hoorn D. E. Boelens P. G. van Norren K. van Leeuwen P. A. (2001). Flavonoids: a review of probable mechanisms of action and potential applications. Am. J. Clin. Nutr. 74, 418–425. 10.1093/ajcn/74.4.418 11566638

[B36] Ohsawa I. Ishikawa M. Takahashi K. Watanabe M. Nishimaki K. Yamagata K.. (2007). Hydrogen acts as a therapeutic antioxidant by selectively reducing cytotoxic oxygen radicals. Nat. Med. 13, 688–694. 10.1038/nm1577 17486089

[B37] Ohta S. (2012). Molecular hydrogen is a novel antioxidant to efficiently reduce oxidative stress with potential for the improvement of mitochondrial diseases. Biochim. Biophys. Acta. 1820, 586–594. 10.1016/j.bbagen.2011.05.006 21621588

[B38] Oms-Oliu G. Rojas-Graü M. A. González L. A. Varela P. Soliva-Fortuny R. Hernando M. I. H. (2010). Recent approaches using chemical treatments to preserve quality of fresh-cut fruit: a review. Postharvest Biol. Technol. 57, 139–148. 10.1016/j.postharvbio.2010.04.001

[B39] Pascual-Teresa S. D. Sanchez-Ballesta M. T. (2008). Anthocyanins: from plant to health. Phytochem. Rev. 7, 281–299. 10.1007/s11101-007-9074-0

[B40] Peng H. Yang T. B. Whitaker B. D. Shangguan L. F. Fang J. G. (2016). Calcium/calmodulin alleviates substrate inhibition in a strawberry UDP-glucosyltransferase involved in fruit anthocyanin biosynthesis. BMC Plant Biol. 16:197. 10.1186/s12870-016-0888-z 27609111 PMC5017016

[B41] Ren S.-C. Sun J.-T. (2014). Changes in phenolic content, phenylalanine ammonia-lyase (PAL) activity, and antioxidant capacity of two buckwheat sprouts in relation to germination. J. Funct. Foods 7, 298–304. 10.1016/j.jff.2014.01.031

[B42] Rohde A. Morreel K. Ralph J. Goeminne G. Hostyn V. de Rycke R.. (2004). Molecular phenotyping of the *pal1* and *pal2* mutants of *Arabidopsis thaliana* reveals far-reaching consequences on phenylpropanoid, amino acid, and carbohydrate metabolism. Plant Cell 16, 2749–2771. 10.1105/tpc.104.023705 15377757 PMC520969

[B43] Santosbuelga C. Mateus N. De F. V. (2014). Anthocyanins. Plant pigments and beyond. J. Agric. Food Chem. 62, 6879–6884. 10.1021/jf501950s 24970106

[B44] Schulz P. Herde M. Romeis T. (2013). Calcium-dependent protein kinases: hubs in plant stress signaling and development. Plant Physiol. 163, 523–530. 10.1104/pp.113.222539 24014579 PMC3793034

[B45] Shi M. Z. Xie D. Y. (2010). Features of anthocyanin biosynthesis in pap1-D and wild-type Arabidopsis thaliana plants grown in different light intensity and culture media conditions. Planta 231, 1385–1400. 10.1007/s00425-010-1142-9 20309578

[B46] Shin D. H. Choi M. G. Lee H. K. Cho M. Choi S. B. Choi G.. (2013). Calcium dependent sucrose uptake links sugar signaling to anthocyanin biosynthesis in Arabidopsis. Biochem Biophys. Res Commun. 430, 634–639. 10.1016/j.bbrc.2012.11.100 23220235

[B47] Su N. Wu Q. Liu Y. Cai J. Shen W. Xia K.. (2014). Hydrogen-rich water reestablishes ROS homeostasis but exerts differential effects on anthocyanin synthesis in two varieties of radish sprouts under UV-A irradiation. J. Agric. Food Chem. 62, 6454–6462. 10.1021/jf5019593 24955879

[B48] Sudha G. Ravishankar G. A. (2003). The role of calcium channels in anthocyanin production in callus cultures of *Daucus carota*. Plant Growth Regul. 40, 163–169. 10.1023/A:1024298602617

[B49] Toufektsian M. C. de Lorgeril M. Nagy N. Salen P. Donati M. B. Giordano L.. (2008). Chronic dietary intake of plant-derived anthocyanins protects the rat heart against ischemia-reperfusion injury. J. Nutr. 138, 747–752. 10.1093/jn/138.4.747 18356330

[B50] Vandesompele J. de Preter K. Pattyn F. Poppe B. Van Roy N. de Paepe A.. (2002). Accurate normalization of real-time quantitative RT-PCR data by geometric averaging of multiple internal control genes. Genome Biol. 3:research0034.1. 10.1186/gb-2002-3-7-research0034 12184808 PMC126239

[B51] Vitrac X. Larronde F. Krisa S. Decendit A. Deffieux G. Mérillon J.-M. (2000). Sugar sensing and Ca^2+^-calmodulin requirement in *Vitis vinifera* cells producing anthocyanins. Phytochemistry 53, 659–665. 10.1016/S0031-9422(99)00620-2 10746878

[B52] Wang C. Q. Zhang Y. F. Tao L. (2005). Activity changes of calmodulin and Ca^2+^-ATPase during low-temperature-induced anthocyanin accumulation in *Alternanthera bettzickiana*. Physiol. Plantarum 124, 260–266. 10.1111/j.1399-3054.2005.00513.x

[B53] Wang X. K. Yang R. Q. Jin X. L. Shen C. Zhou Y. L. Chen Z. J. (2016). Effect of supplemental Ca^2+^ on yield and quality characteristics of soybean sprouts. Sci. Hortic. 198, 352–362. 10.1016/j.scienta.2015.11.022

[B54] Wu Q. Su N. Cai J. Shen Z. Cui J. (2015). Hydrogen-rich water enhances cadmium tolerance in Chinese cabbage by reducing cadmium uptake and increasing antioxidant capacities. J. Plant Physiol. 175, 174–182. 10.1016/j.jplph.2014.09.017 25543863

[B55] Xia X. Ling W. Ma J. Xia M. Hou M. Wang Q.. (2006). An anthocyanin-rich extract from black rice enhances atherosclerotic plaque stabilization in apolipoprotein E–deficient mice. J. Nutr. 136, 2220–2225. 10.1093/jn/136.8.2220 16857844

[B56] Xie Y. Chen P. Yan Y. Bao C. Li X. Wang L.. (2017). An atypical R2R3 MYB transcription factor increases cold hardiness by CBF-dependent and CBF-independent pathways in apple. New Phytol. 218, 201–218. 10.1111/nph.14952 29266327

[B57] Xie Y. J. Mao Y. Lai D. W. Zhang W. Shen W. B. (2012). H_2_ enhances Arabidopsis salt tolerance by manipulating ZAT10/12-mediated antioxidant defence and controlling sodium exclusion. PLoS ONE 7:e49800 10.1371/journal.pone.0049800 23185443 PMC3504229

[B58] Xie Y. J. Zhang W. Duan X. L. Dai C. Zhang Y. H. Cui W. T. (2015). Hydrogen-rich water-alleviated ultraviolet-B-triggered oxidative damage is partially associated with the manipulation of the metabolism of (iso)flavonoids and antioxidant defence in *Medicago sativa*. Funct. Plant Biol. 42, 1–10. 10.1071/FP15204 32480752

[B59] Xie Y. Tan H. J. Ma Z. X. Huang J. R. (2016). DELLA proteins promote anthocyanin biosynthesis via sequestering MYBL2 and JAZ suppressors of the MYB/bHLH/WD40 complex in *Arabidopsis thaliana*. Mol. Plant 9, 711–721. 10.1016/j.molp.2016.01.014 26854848

[B60] Xu W. Dubos C. Lepiniec L. (2015). Transcriptional control of flavonoid biosynthesis by MYB–bHLH–WDR complexes. Trends Plant Sci. 20, 176–185. 10.1016/j.tplants.2014.12.001 25577424

[B61] Xu W. P. Peng H. Yang T. B. Whitaker B. Huang L. H. Sun J. H.. (2014). Effect of calcium on strawberry fruit flavonoid pathway gene expression and anthocyanin accumulation. Plant Physiol. Biochem. 82, 289–298. 10.1016/j.plaphy.2014.06.015 25036468

[B62] Xu Y. Y. Zhu X. W. Gong Y. Q. Xu L. Wang Y. Liu L. W. (2012). Evaluation of reference genes for gene expression studies in radish (*Raphanus sativus* L.) using quantitative real-time PCR. Biochem. Biophys. Res. Commun. 424, 398–403. 10.1016/j.bbrc.2012.06.119 22771808

[B63] Xu Z. Mahmood K. Rothstein S. J. (2017). ROS induces anthocyanin production via late biosynthetic genes and anthocyanin deficiency confers the hypersensitivity to ROS-generating stresses in Arabidopsis. Plant Cell Physiol. 58, 1364–1377. 10.1093/pcp/pcx073 28586465

[B64] Yan J. W. Guan L. Sun Y. Zhu Y. Liu L. Lu R.. (2015). Calcium and ZmCCaMK are involved in brassinosteroid-induced antioxidant defense in maize leaves. Plant Cell Physiol. 56, 883–896. 10.1093/pcp/pcv014 25647327

[B65] Yang T. B. Poovaiah B. W. (2003). Calcium/calmodulin-mediated signal network in plants. Trends Plant Sci. 8, 505–512. 10.1016/j.tplants.2003.09.004 14557048

[B66] Yoo S. D. Cho Y. H. Sheen J. (2007). Arabidopsis mesophyll protoplasts: a versatile cell system for transient gene expression analysis. Nat. Protoc. 2, 1565–1572. 10.1038/nprot.2007.199 17585298

[B67] Zhang J. L. Chen C. S. Zhang D. Li H. H. Li P. M. Ma F. W. (2014). Reactive oxygen species produced via plasma membrane NADPH oxidase regulate anthocyanin synthesis in apple peel. Planta 240, 1023–1035. 10.1007/s00425-014-2120-4 25000919

[B68] Zhang W. H. Rengel Z. Kuo J. (1998). Determination of intracellular Ca^2+^ in cells of intact wheat roots: loading of acetoxymethyl ester of Fluo-3 under low temperature. Plant J. 15, 147–151. 10.1046/j.1365-313X.1998.00188.x

[B69] Zhang X. Zhao X. Wang Z. Shen W. Xu X. (2015). Protective effects of hydrogen-rich water on the photosynthetic apparatus of maize seedlings (*Zea mays* L.) as a result of an increase in antioxidant enzyme activities under high light stress. Plant Growth Regul. 77, 43–56. 10.1007/s10725-015-0033-2

[B70] Zhi H. H. Liu Q. Q. Xu J. Dong Y. Liu M. P. Zong W. (2017). Ultrasound enhances calcium absorption of jujube fruit by regulating the cellular calcium distribution and metabolism of cell wall polysaccharides. J. Sci. Food Agric. 97, 5202–5210. 10.1002/jsfa.8402 28447385

[B71] Zhu Y. C. Liao W. B. Niu L. J. Wang M. Ma Z. J. (2016). Nitric oxide is involved in hydrogen gas-induced cell cycle activation during adventitious root formation in cucumber. BMC Plant Biol. 16:146. 10.1186/s12870-016-0834-0 27352869 PMC4924243

