# Supplementary material for: Increased Cytosolic Calcium Contributes to Hydrogen-Rich Water-Promoted Anthocyanin Biosynthesis Under UV-A Irradiation in Radish Sprouts Hypocotyls
Source: Front Plant Sci. 2018 Jul 16;9:1020. doi: 10.3389/fpls.2018.01020 (PMC6055044; doi:10.3389/fpls.2018.01020)
Supplement: Supplementary file 1 [file Table_1.DOCX]

**Table S1** The sequences of primers for qPCR.

| Gene | Accession No. | Forward primer（5’→3’） | Reverse primer（5’→3’） |
| --- | --- | --- | --- |
| *RsPAL* | XM_018603499 | GATGTAGACGCTATGTTATGC | CCTTCACCATTCTCTTCACT |
| *RsCHS* | XM_018591340 | AACTCACTCTTCTGGATAGC | CTCATCTTCTCCGCCTTG |
| *RsCHI* | XM_018636123 | GCTTCCATCCTCTTCGCTCTC | ACTGCCTCTGCCAACAACTTA |
| *RsF3H* | XM_018577879 | ATGGCTCCAGGAACTCTAACC | CCGTGCTGAACTCATTGTAGG |
| *RSF3’H* | XM_018587404 | TTGTGGTTGCCGCCTCTAA | TTCTCCATCGTTGTCCATAAGG |
| *RsDFR* | XM_018599434 | TTCGTCCGTGCCACTGTT | GCGTCATCGTAGCTTCCTTC |
| *RsLDOX* | XM_018622460 | ACAAGACGGTCCTCAAGTCC | CCAGTCCATAGCAGCCTTCT |
| *RsUFGT* | XM_018594912 | GTAGCGATTAAGCCTCCTT | CCAGCCTCCATACACAAC |
| *RsPAP1* | XM_018613241 | CTGTAGACTAAGATGGTTGAAC | ATGAAGGCGAAGAAGAAGAT |
| *RsPAP2* | XM_018635639 | TAGGTGTAGGAAGAGTTGTAG | ATGAAGACGGAGAAGAAGAT |
| *RsACTIN2* | XM_018637077 | TCGTGACCTTACTGATTACC | CTCCATCTCCTGCTCGTA |
| *RsTEF2* | XM_018589947 | GGAGCTAAGCCTGAGCACTT | AGGGCCGAAGCAAAGCTTAC |

**Materials and methods**

**Measurement of H_2_ Content**

The H_2_ release was measured using a needle-type Hydrogen Sensor (Unisense, Denmark) according to Xie et al. (2014) and the manufacturer’s instructions. The H_2_-specific electrode had was polarized overnight prior to use. The radish seedlings were placed on 1.5% agarose gel pieces to avoid damage to the electrode tip. When performing electrode analysis, this needle punctured leaf tissues (about 200 μm below the hypocotyl surface, the tip was able to move up and down accurately in 50-μm steps as controlled by a micromanipulator equipped with a binocular microscope). After the basal line of H_2_ signal was stable, treatment solution was added to immerse the electronic tip. Corresponding data were then recorded. As a result, the released H_2_, which is supposed to be dissolved in extracellular fluid of radish hypocotyl tissues, was measured. A standard solution of HRW was prepared by saturating H_2_ gas in distilled water (781 μM at 25°C) at atmospheric pressure, while distilled water was used as a negative control. All manipulations were performed at 25°C.


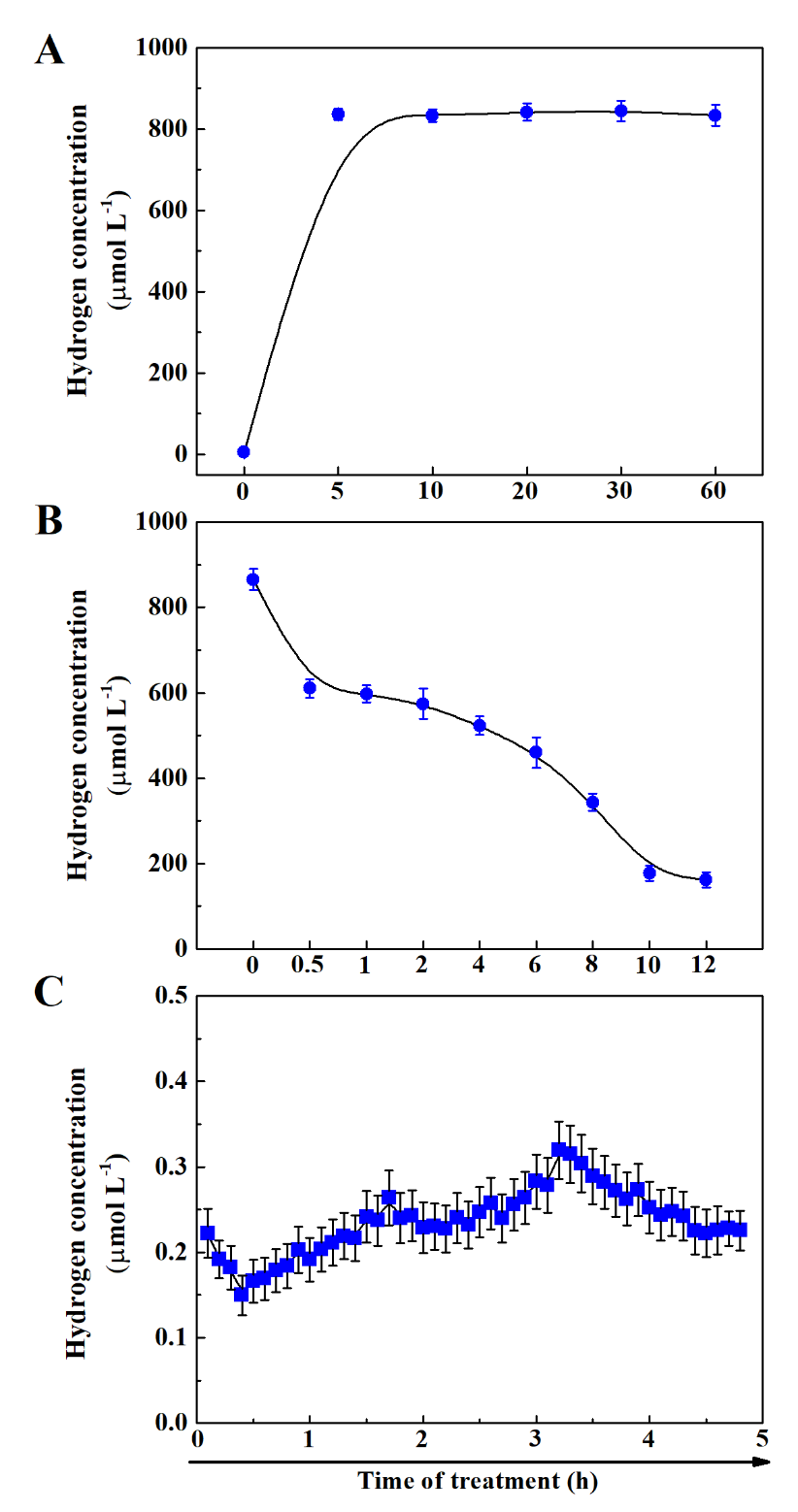


**Figure S1** The changes of H_2_ concentration in water with the bubbling time (A) in our study. The changes of H_2_ concentration in HRW with releasing time (B). The real-time dynamics of H_2_ concentration in the hypocotyls of radish sprouts with the treatment time (C). Values are the means ± SE of three independent experiments with at least three replicates for each.


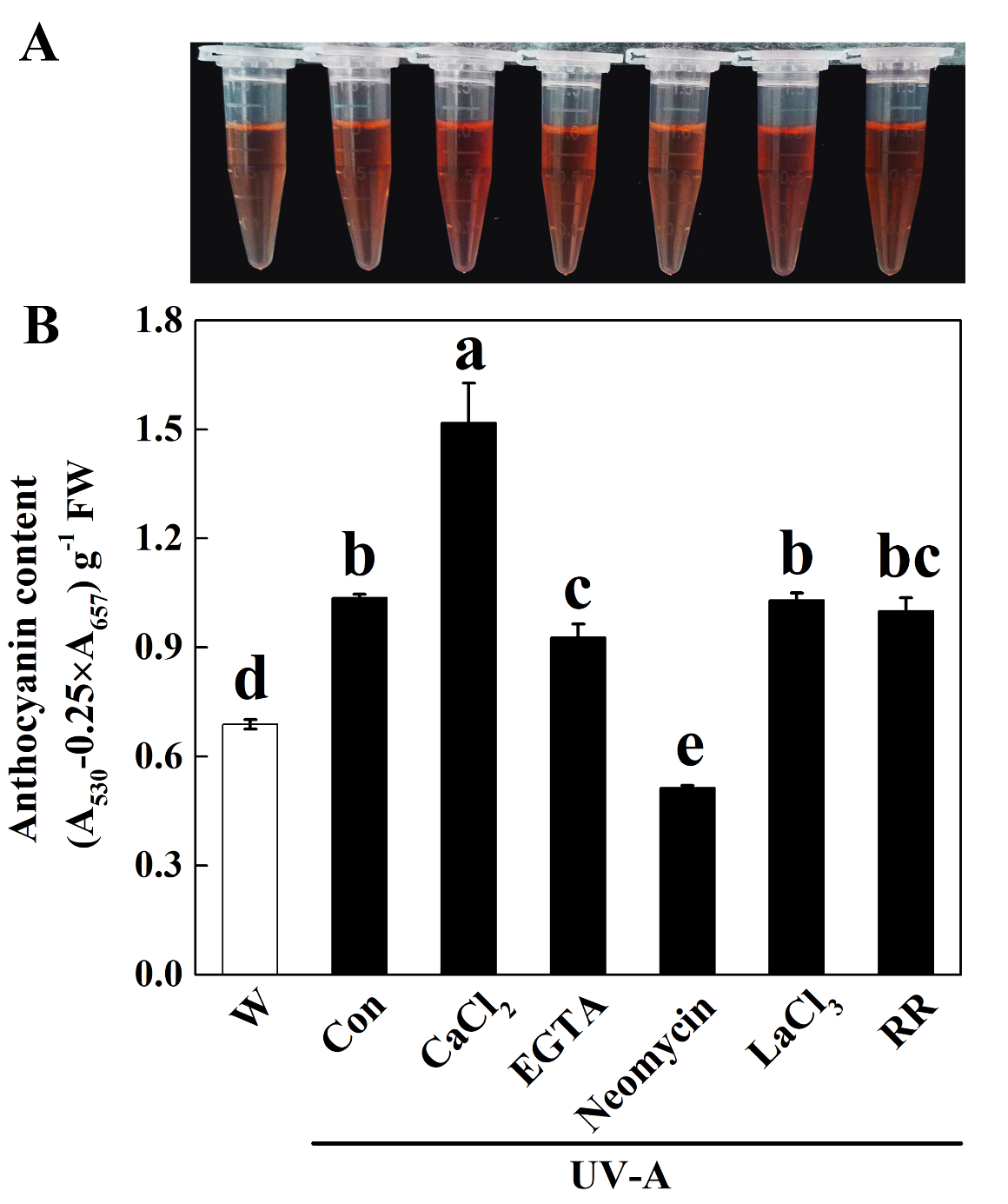


**Figure S2** Effects of CaCl_2_ and calcium antagonists on anthocyanin content improved by HRW under UV-A. Radish sprouts were grown in the dark for 36 h and then exposed to LED white light (W) or UV-A irradiation. Radish sprouts cultured under UV-A were treated with 10 mM CaCl_2_, 1 mM EGTA,1 mM Neomycin, 5 mM LaCl_3_ and 70 μM RR, respectively. Anthocyanin content was measured at 48 h plant growth. Data are presented as means ± SE of three independent experiments with at least three replicates for each. Bars with different letters are significantly different at the *P* < 0.05 level according to Duncan’s multiple comparison.


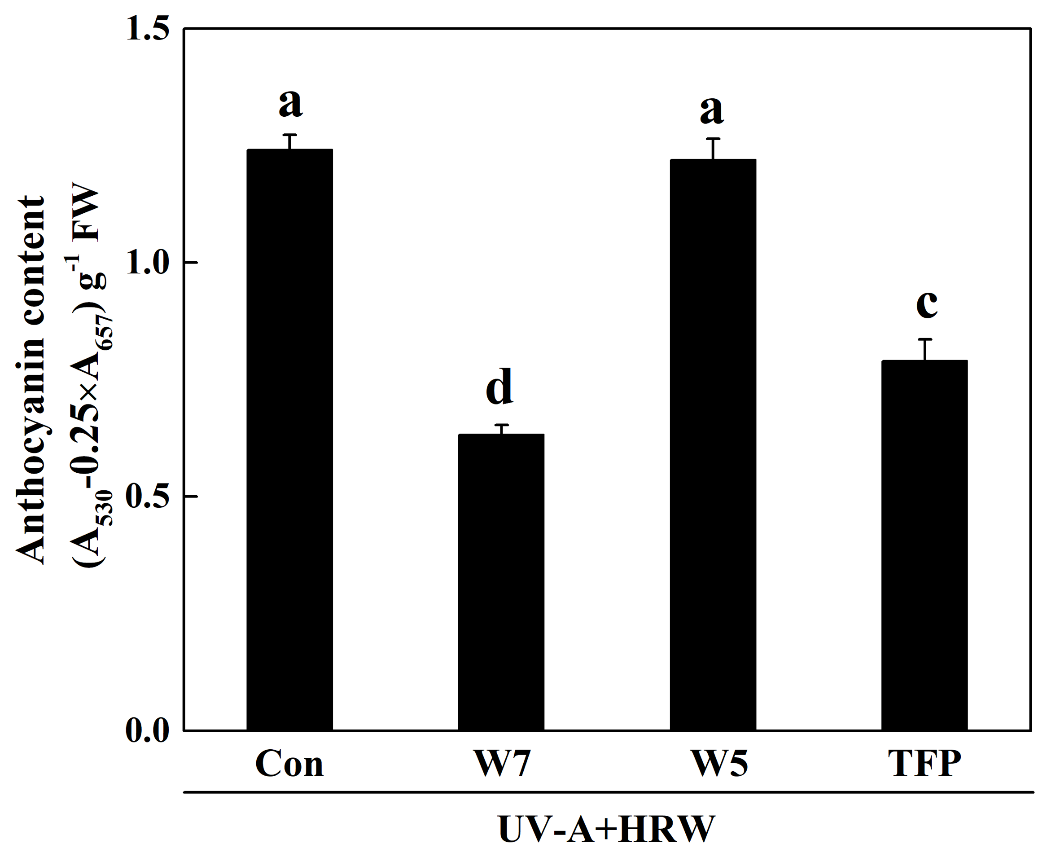


**Figure S3**. Effects of CaM antagonists on anthocyanin content improved by HRW under UV-A. Radish sprouts were grown in the dark for 36 h and then cultured in solution containing HRW (100% saturation) under UV-A. Radish sprouts were treated with 150 μM W7, 150 μM W5 or 100 μM TFP, respectively. The anthocyanin content was measured at 48 h plant growth. Data are presented as means ± SE of three independent experiments with at least three replicates for each. Bars with different letters are significantly different at the *P* < 0.05 level according to Duncan’s multiple comparison.
